# A CoAP-Based Network Access Authentication Service for Low-Power Wide Area Networks: LO-CoAP-EAP

**DOI:** 10.3390/s17112646

**Published:** 2017-11-17

**Authors:** Dan Garcia-Carrillo, Rafael Marin-Lopez, Arunprabhu Kandasamy, Alexander Pelov

**Affiliations:** 1 Department Information and Communication Engineering (DIIC), Faculty of Computer Science, University of Murcia, 30100 Murcia, Spain; rafa@um.es; 2 Acklio, 2 BIS rue de la Chataigneraie, 35576 Cesson-Sevigne, France; arun@ackl.io (A.K.); a@ackl.io (A.P.); 3 Institut MINES TELECOM, TELECOM Bretagne, 2 rue de la Chataigneraie CS 17607, 35576 Cesson-Sevigne CEDEX, France

**Keywords:** lightweight, network access authentication, IoT, LP-WAN, CoAP, EAP, AAA

## Abstract

The Internet-of-Things (IoT) landscape is expanding with new radio technologies. In addition to the Low-Rate Wireless Personal Area Network (LR-WPAN), the recent set of technologies conforming the so-called Low-Power Wide Area Networks (LP-WAN) offers long-range communications, allowing one to send small pieces of information at a reduced energy cost, which promotes the creation of new IoT applications and services. However, LP-WAN technologies pose new challenges since they have strong limitations in the available bandwidth. In general, a first step prior to a smart object being able to gain access to the network is the process of network access authentication. It involves authentication, authorization and key management operations. This process is of vital importance for operators to control network resources. However, proposals for managing network access authentication in LP-WAN are tailored to the specifics of each technology, which could introduce interoperability problems in the future. In this sense, little effort has been put so far into providing a wireless-independent solution for network access authentication in the area of LP-WAN. To fill this gap, we propose a service named Low-Overhead CoAP-EAP (LO-CoAP-EAP), which is based on previous work designed for LR-WPAN. LO-CoAP-EAP integrates the use of Authentication, Authorization and Accounting (AAA) infrastructures and the Extensible Authentication Protocol (EAP) protocol. For this integration, we use the Constrained Application Protocol (CoAP) to design a network authentication service independent of the type of LP-WAN technology. LO-CoAP-EAP represents a trade-off between flexibility, wireless technology independence, scalability and performance in LP-WAN.

## 1. Introduction

In the last few years, the global information network consisting of Internet-connected objects known as the Internet of Things (IoT) [1] has experienced an important growth. Several wireless technologies, such as Bluetooth [2] or IEEE 802.15.4 [3], give support to the myriad of devices connected to IoT networks. These wireless technologies typically offer a short-medium range coverage and allow the creation of Low-Rate Wireless Personal Area Networks (LR-WPAN). In addition to the aforementioned wireless technologies, there has been a recent impulse toward Low-Power Wide Area Networks (LP-WAN) [4]. LP-WAN encompasses wireless technologies for long-range communications. LP-WAN allows one to send small pieces of information between the IoT devices and an antenna up to several kilometers with small energy consumption, expanding the impact of IoT applications to a larger scale. Among such applications, we can mention the connection of alarms to the cloud, smart farming and precision agriculture [4]. As a downside, LP-WAN only supports data rates as low as 50 b/s or 250 kB/s, frame sizes as low as eight bytes and restrictions to access the medium with a duty cycle of 0.1% to 10% on some Industrial, Scientific and Medical (ISM) bands [5]. In other words, apart from the constraints observed in LR-WPAN, LP-WAN adds a very constrained communication link (beyond those observed in LR-WPAN). Due to the concept around LP-WAN being promising, some research groups and alliances (e.g., LoRaWAN [6], Sigfox [7], Developers’ Alliance for Standards Harmonization of ISO 18000-7 (DASH7)-7 [8], Wireless Smart Ubiquitous Network (WI-SUN) [9], etc.) are putting their resources into defining their own specific solutions to cover the downsides of the LP-WAN networks quickly. Very recently, the Internet Engineering Task Force (IETF) has created a Low-Power Wide Area Networks (lpwan) Working Group to determine how they can contribute to the evolution of LP-WAN by defining technology independent protocols and solutions that favor interoperability between the different wireless technologies involved in LP-WAN. For example, modifications to the 6LoWPAN standard are under consideration to adapt IPv6 to LP-WAN [10]. Another example is the adaptation of the Constrained Application Protocol (CoAP) [11] to LP-WAN. CoAP is a specialized web transfer protocol for constrained nodes and networks designed to implement different types of services (e.g., sensor readings, remote configuration, etc.) in IoT. While there is an effort for homogenizing communications in this type of network based on IPv6 and CoAP, little has been done to design a unifying solution, providing a common and well-defined model for network access authentication for LP-WAN networks, independent of the wireless technology. Nevertheless, this process, which involves authentication, authorization and key management, is also fundamental since it allows operators and network administrators to decide whether a particular device can or cannot join the network.

In this sense, the LPWAN WG has discussed the use of the Authentication Authorization and Accounting (AAA) infrastructures to support network access authentication [5,12,13,14]. The reason is that LP-WAN networks look very similar to current cellular deployments, but with very limited resources. In fact, current wireless networks such as 3G, WiMAX or WiFi use, traditionally, AAA infrastructures to control the access to the network service at a large scale [15,16,17]. These infrastructures have been based on two main protocols: Remote Authentication Dial-In User Service (RADIUS) [18] or Diameter [19]. In conjunction with AAA, the Extensible Authentication Protocol (EAP) [20] is also considered to authenticate the devices because it provides a wireless technology independent protocol for flexible authentication and robust key management [21]. By definition, the EAP key management framework allows obtaining key material to derive new key material to bootstrap a security association in a concrete wireless technology.

In this paper, we propose a first solution for network access authentication in LP-WAN called Low-Power CoAP-EAP (LO-CoAP-EAP) based on three pillars: AAA, EAP and CoAP. The use of AAA infrastructures provides scalability and roaming for the network access authentication; EAP allows different smart objects and organizations to use different authentication mechanisms (based on symmetric keys, certificates, etc.) depending on their requirements; and CoAP, which is used as a constrained transport for EAP between the smart object and the network, both connected through a link with a very limited bandwidth. The general design of LO-CoAP-EAP starts from our previous work in [22], named CoAP-EAP, but is redesigned to take into account the constraints in LP-WAN. In particular, LO-CoAP-EAP provides a CoAP-based service for network access authentication in LP-WAN, which promotes homogeneity and independence between different wireless technologies. The new design of LO-CoAP-EAP reduces the number of exchanges and the overall number of bytes sent. As a collateral effect, we will also prove that LO-CoAP-EAP substantially improves CoAP-EAP in LR-WPAN networks.

The rest of this paper is organized as follows. Section 2 gives the background in order to understand our solution. Section 3 details the related work. Section 4 details the proposed network access authentication service, LO-CoAP-EAP: the architecture and operation. In Section 5, we show a performance evaluation including message length, authentication time, the proportion of successful authentications (success ratio) and the energy consumption in two test-beds: (1) a real deployment of a LP-WAN network based on LoRa technology to get in-the-field measurements; (2) a network based on 6LoWPAN using the Cooja simulator to also show the impact in LR-WPAN networks. With these two test-beds, we have compared LO-CoAP-EAP against existing related work. Finally, we provide some conclusions and future work lines in Section 6.

## 2. Background

### 2.1. Authentication, Authorization and Accounting Framework

The Authentication, Authorization and Accounting (AAA) Framework [23] supports three basic security services used in network deployments: authentication, to establish the identity of the end user; authorization, describing the conditions under which the user is able to access the network resources; accounting, tracking the resources consumed by the user. The AAA framework defines a model consisting of an End User (EU) who intends to gain access to some specific network service, an Identity Provider (IdP) that stores the identity of the EU and some long-term credentials; and a Service Provider (SP) that manages the access to the network service. In a non-federated scenario, the IdP and the SP belong to the same organization (the IdP’s organization). In a federated scenario, where the IdP and SP belong to different organizations, some bilateral agreements are assumed among the domains conforming the federation. The participant organizations in the federation will have independent AAA servers that will be able to communicate and exchange AAA information among them using an AAA protocol. The most commonly deployed AAA protocols are Diameter [19], widely deployed in 3G networks, and RADIUS [18], used in WiFi and WiMAX.

The SP operates the Network Access Server (NAS) that communicates with the user (e.g., a smart object) and the AAA infrastructure. In the most simple scenario, an AAA infrastructure consists of a NAS, with a direct connection to the AAA server. In more complex scenarios, additional AAA servers (called AAA proxies) can be deployed between the NAS and the AAA server for scalability or to support federated access.

### 2.2. Extensible Authentication Protocol

The Extensible Authentication Protocol (EAP) [20] is a standard protocol for authentication that allows the execution of authentication mechanisms (e.g., based on digital certificates, symmetric keys, etc.), named EAP methods, without changing the protocol. As an example of the EAP method, we can mention EAP-PSK [24], which provides a lightweight authentication mechanism based on Pre-Shared Key (PSK). Other examples of EAP methods, such as EAP-Transport Layer Security (EAP-TLS) , can be seen in [25].

EAP has been designed with the principle of media independence. That is, the protocol is independent of the wireless technology used. A different matter is that EAP is not independent of the link-layer and that is why the characteristics of a protocol that intends to transport EAP messages (EAP lower layer) is specified in EAP.

By definition, EAP is a lock-step protocol, which means it handles a single packet, a request or a response, per flight. Each EAP request is answered with an EAP response. The number of message exchanges depends on the EAP method used. This gives flexibility to choose the authentication that fits best in each case. Every EAP method is run between the EAP peer and the EAP server through the EAP authenticator acting as a forwarder. To start an EAP authentication, the EAP authenticator typically sends an EAP request/identity message to the EAP peer, whom in turn answers with its identity. The identity is sent following the Network Access Identifier (NAI) format [26] (e.g., smartobject@homedomain). The NAI contains the smart object identity, separating the domain name (homedomain) with an @ sign. Once the EAP server receives the identity of the EAP peer, it is able to select the EAP method to be performed. The EAP method is performed using EAP request/responses between the EAP server and the EAP peer.

There are two models to deploy EAP. On the one hand, we have the EAP standalone model, in which the EAP authenticator and the EAP server are co-located in the same device. This model can be easily used in small deployments, where no AAA is required. On the other hand, when scalability becomes a must (as happens in the context of this paper), the EAP pass-through authenticator model is used. In this case, the communication between the EAP pass-through authenticator and the EAP server is done using an AAA protocol. Common to both cases is the use of an EAP lower layer to carry EAP messages between the EAP peer and the EAP authenticator. In this paper, we have designed a CoAP-based EAP lower layer and, therefore, independent of the EAP method in use.

One important feature to take into account is that some EAP methods [25] generate key material. According to the EAP Key Management Framework (KMF) [21], two keys are exported after a successful EAP authentication: the Master Session Key (MSK) and the Extended Master Session Key (EMSK). From both keys, only the MSK has a defined use for network access authentication in order to run a Security Association Protocol (SAP) to derive Transient Session Keys (TSK). In turn, the TSKs allow one to protect the communications between the EAP peer and EAP authenticator. The MSK is sent by the AAA server to the EAP authenticator using the AAA protocol, while the EMSK must not be provided to any other entity, keeping it only between the EAP peer and the EAP server.

### 2.3. Constrained Application Protocol

CoAP is defined in [11] as a web transfer protocol specifically designed to be used with constrained devices and constrained networks in the IoT. The purpose of CoAP is to realize a subset of REST optimized for M2M applications. In fact, it has some built-in capabilities designed for M2M such as discovery of services and capabilities, support for multicast and asynchronous message exchanges, with a very low overhead and simplicity, having constrained environments in mind. In the specific case of LP-WAN, CoAP presents a proper alternative where communication between devices has to be performed in a standardized manner using a RESTful protocol, with low overhead. Recently, a work has been proposed to further reduce the overhead of CoAP by Minaburo et al. [10].

CoAP distinguishes three different categories of messages: request, response and empty. A message can be of four different types: Confirmable (CON), Non-confirmable (NON), Acknowledgment (ACK) and Reset (RST). Unlike NON messages, CON messages require the reception of an ACK. RST is used when the received message (CON or NON) cannot be processed. CoAP messages contain a code field, which gives semantics to the message, whether it is a request or a response, and if the response denotes success or failure of some kind. There is another field called token, which is used to match requests with responses. Additionally, CoAP supports the addition of a sequence of zero or more CoAP Options in the Type-Length-Value (TLV) format. CoAP requests contain different methods (or actions over the resource): GET, POST, PUT and DELETE. GET obtains the current content of the resource identified by the URI. POST messages allow for the client and server to exchange information and, according to the context of the information, to process the request accordingly. PUT is used to create or update the resource identified by the URI. Finally, DELETE requests that the resource represented by the URI has to be deleted.

## 3. Related Work

Network access authentication for IoT is described in Heer et al. [27] and Garcia-Morchon et al. [28] as part of the process of a smart object joining an IoT network securely, at a specific time and place. This process entails the authentication and authorization of the smart object, as well as the transfer of security parameters (e.g., key material) for a trustworthy operation in the security domain.

In general, although most of the current work for network access authentication in IoT has been devoted to LR-WPAN, some of these (specified below) deserve attention due to the relation to our solution. Large-scale scenarios use AAA infrastructures and EAP, while small to medium scale do not involve AAA infrastructures. For example, Garcia-Morchon et al. [28] in the case of the centralized scenario point out EAP as a candidate to perform the authentication and generation of key material, proposing the Protocol for carrying Authentication for Network Access (PANA) [29] as a standard and link-layer independent EAP lower layer. In fact, the ZigBee IP standard [30] uses PANA as a protocol for network access authentication. As such, our proposal LO-CoAP-EAP can be compared with PANA, as we will analyze in Section 5. O’Flynn et al. [31] consider also a centralized architecture using EAP and PANA or 802.1X [32] as EAP lower layers, while Sarikaya [33] and Sarikaya et al. [34] propose EAP-TLS concretely as the authentication protocol, but without interaction with an AAA infrastructure. S. Das et al. [35] also propose a centralized alternative using PANA, EAP and AAA to bootstrap a PSK to establish a Datagram Transport Layer Security (DTLS) [36] or Internet Key Exchange (IKEv2) [37] security association between the smart object and the PANA Agent (PAA), using afterwards CoAP for the normal operation. Alternatively, Moreno et al. [38] designed and implemented a lightweight version of a PANA client (PaC) for Contiki O.S. [39] (PANATIKI) by adapting PANA for constrained devices. Finally, Garcia-Carrillo and Marin-Lopez [22] proposes a technology independent EAP lower layer using CoAP to transport EAP messages (CoAP-EAP) that improves the PANA-based solutions, to leverage the features of CoAP as a suitable protocol for communication between constrained devices and use of AAA infrastructures to provide scalability and federation capabilities.

There are also solutions that use EAP adapted to IoT, by either modifying EAP itself, or defining new EAP methods more optimized for the constraints of IoT networks. However, these solutions imply that wireless technologies where EAP is applied need to define an EAP lower layer specific to the technology. For example, it would imply that existing LP-WAN technologies should design their own link-layer EAP lower layers to transport those EAP methods, which is far from the general case. On the contrary, LO-CoAP-EAP offers a wireless independent EAP lower layer to avoid this problem and allows existing or new EAP methods for IoT to be used without modifying the underlying technology.

Thus, the constraint LP-WAN imposes on the network link requires adapting or redesigning existing solutions. Most of the LP-WAN technologies are fairly recent and are still under development, and as a consequence, solutions related to security and network access authentication are still immature. Specifically, some of the alliances and organizations working in the area of LP-WAN propose solutions to authenticate and secure the communications using pre-shared keys. Additionally, the key management is usually handled in a static manner, with no dynamic key distribution, and there is little information about how the necessary keys will be managed, installed, refreshed, etc., or how the devices will be authenticated to access the network and how they will interact securely with the infrastructure. For example, DASH-7 [8] includes the notion of authentication and access control, defining three users (root, user and guest) and authentication keys root and user, as well as a network key. There is no specific mention about the method used to generate the aforementioned keys, if they are configured manually, or provided dynamically using other methods of key management. Sigfox [7] provides integrity of the communications, with encryption at the application level, and it considers the future integration of a secure element to store key material. However, there is no mention of how these keys are installed, either manually or through dynamic key management methods. IEEE 802.11ah [40] defines a modification in the scheduling of the network access authentication over the IEEE 802.11ai amendment, but no references to the modifications of the network access authentication methods are made, so we consider that they are the same as defined for IEEE 802.11. LoRaWAN [6] does include a join procedure to authenticate the IoT devices based on pre-shared key Application Session Key (AppSKey) to derive fresh keys (NetworkSKey and AppSKey) to protect the LoRa link between the IoT device and the network. Recent work ([13] and Diameter [14]) has defined a way to integrate the LoRaWAN join procedure with AAA infrastructures to provide scalability and roaming support. However, these solutions are tailored to LoRaWAN.

Table 1 summarizes the current state of several technologies related to LP-WAN security and network access authentication, showing the algorithms used to provide integrity and encrypt the messages and if the technology provides a key management protocol to derive fresh key material to protect the link. There is a common occurrence of the use of symmetric cryptography, understandable due to its properties, providing security at a computationally low cost in comparison with asymmetric cryptography, and furthermore, the existence of hardware implementations of the most common crypto suite, Advanced Encryption Standard (AES), increases its efficiency.

Thus, to the best of our knowledge, this paper is the first work to contribute a solution for network access authentication for LP-WAN that tries to deal with the process in a homogeneous way through different technologies.

## 4. Network Access Authentication in LP-WAN: LO-CoAP-EAP

### 4.1. Requirements of the Service

The general requirement that leads to the design of the LO-CoAP-EAP service is to have a network access authentication service for LP-WAN regardless of the underlying radio technology being used, relaying on current standards, with the following characteristics:

- Flexible authentication mechanism: The service has to provide the needed flexibility for the authentication due to the variety of operators, technologies and requirements of each deployment.
- Operational homogeneity: The service had to be built on top of a protocol that is common to most of the IoT devices.
- Link-layer independent solution: The solution should be independent of the link-layer technology to be supported by the set of LP-WAN technologies.
- Reduce overhead: Due to the high restrictions of LP-WAN in terms of bandwidth, we need a solution with a reduced overhead, saving as much messages and bytes sent over the link as possible.
- Bootstrapping subsequent security association protocols: As each LP-WAN technology may rely on different protocols to secure its data communications, we need to provide key material for different protocols to secure subsequent communications between the smart object and the network.

### 4.2. The LO-CoAP-EAP Service

Low-Overhead CoAP-EAP (LO-CoAP-EAP) is a CoAP-based and link-layer independent network access authentication service designed considering the constraints imposed by LP-WAN networks. LO-CoAP-EAP builds on top of three main technologies: CoAP, AAA and EAP. We use CoAP as a lightweight application protocol for constrained devices, allowing reusing source code in existing smart objects since CoAP is a common piece in IoT stacks for Machine-to-Machine (M2M) communications [41]. The integration with AAA infrastructures provides scalability, roaming/federation support and a common core independent of the technology to centralize the authentication and key distribution procedures. With EAP, we have flexibility to choose the authentication method, depending on the requirements of different organizations. Additionally, the EAP Key Management Framework (EAP KMF) provides the rules for key derivation to bootstrap security associations for different technologies, to protect data traffic in the constrained link.

Next, we describe the architecture of LO-CoAP-EAP network authentication service and its operation, and we detail the main changes with respect to our previous work in order to adapt the solution to cover LP-WAN networks.

### 4.3. Architecture

LO-CoAP-EAP defines three main entities in its general architecture: the smart object, the controller and the AAA server. The smart object is the target of the authentication, and the controller manages the network. It offers services to the security domain and intermediates during network access authentication between the smart object and the AAA server. Finally, the AAA server is in charge of the authentication and granting permissions for the requested services. LO-CoAP-EAP entities integrate EAP, CoAP and AAA as follows: the smart object instantiates an EAP peer and a CoAP client and server; the controller integrates an EAP authenticator, a CoAP server and client and an AAA client to interact with the AAA server (authentication server) of the identity provider. It is worth noting that there can be one or more AAA servers between the controller and the authentication server.

The Internet Engineering Task Force (IETF)LPWAN WG defines in its overview document [12] a generic terminology for the architecture. They define end-devices as the things, sensors, actuators, etc. The radio gateway is the end-point of the constrained link. The network gateway (or network server) is the interconnection between the radio gateway and the Internet. Finally, LPWAN-AAA is the entity in charge of managing the authentication. The LO-CoAP-EAP architecture maps to their LP-WAN counterparts as follows: the smart object is the end-device; the controller acts as the network server; and the AAA server as the LPWAN-AAA.

### 4.4. General Operation

**Figure 1:**
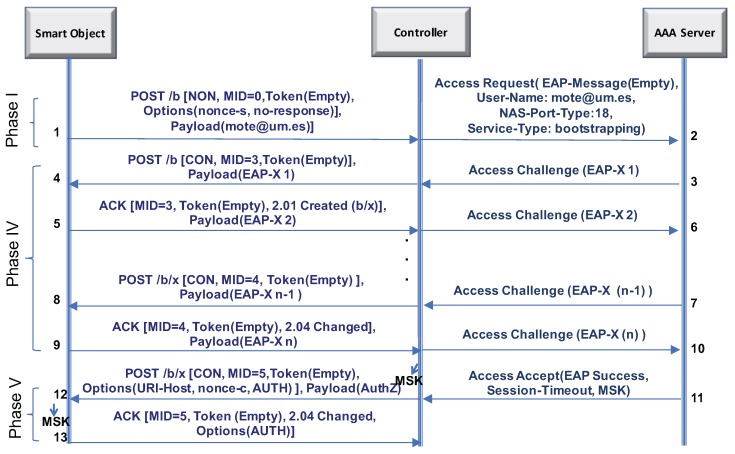
Low-Overhead (LO)-CoAP-EAP bootstrapping service flow using any EAP method X (EAP-X).

To refer to the CoAP-based service for network authentication, LO-CoAP-EAP uses the URI coap://<controller-IP>/b. The smart object, acting as the CoAP client, initiates the LO-CoAP-EAP exchange. Without loss of generality, we use RADIUS as the AAA protocol in the description. The operation, shown in Figure 1, is as follows:

The smart object sends a POST message (Step 1) with the no-response option [42]. This option allows the smart object to signal it does not expect a response to this request. In this first message, the smart object sends a random number nonce-s carried in an option named nonce and the smart object’s identity in NAI format [26] (user@domain.org) in the payload. After this first message, all the remaining exchanges will be done with the controller acting as the CoAP client and the smart object as the CoAP server. This role choice follows the guidelines in [43] to simplify the implementation assuming that the smart object will be more constrained than the controller. The controller can now process the smart object’ identity and send it to the AAA server in a RADIUS access-request (Step 2). Typically, the identity is transported in a EAP response/identity, but this implies sending an EAP request/identity first. To save this exchange, the RADIUS (and Diameter) standard allows the smart object’s identity to be carried into an attribute called user-name instead of EAP-response/identity message. In this case, the attribute EAP-message, which contains EAP messages, will be empty in this message [44,45]. Additionally, it will include a NAS-port-type. This attribute indicates the physical port of the controller that is authenticating the user, and it is useful for the AAA server to know whether the access is being performed over an LP-WAN link, which may modulate the authorization decision and the delivery of configuration parameters for the controller. After the AAA server processes this message, it decides what EAP method is to be used based on the smart object’ identity; let us call it EAP-X. Then, it responds with the first message of the EAP method (EAP-X 1) embedded in the attribute EAP-message of a RADIUS access challenge (Step 3). When this message arrives at the controller (Step 4), it decapsulates EAP-X 1, encapsulating it in the payload of a confirmable POST message. The token value in CoAP is set to empty to further reduce the length of the message. The smart object answers this POST with a piggybacked response that contains the EAP response (EAP-X 2) (Step 5). The controller will then forward EAP-X 2 to the AAA server using a RADIUS access-challenge (Step 6), and so on. This process continues until the EAP method finishes (Steps 7–10), the number of exchanges depending on the EAP method. Finally, the AAA server sends a RADIUS access-accept with an EAP success. Along with the EAP success, the MSK is delivered to the controller with a network access lifetime (i.e., session-timeout) (Step 11). Then, the controller sends a confirmable POST message that contains a nonce nonce-c in the nonce option and authorization data like the session lifetime (Step 12) in the payload.

Typically, the EAP success is also included in this message. However, we do not include it in order to save bandwidth, since the EAP standard allows the EAP peer (smart object) to proceed without receiving the EAP success, but with an alternate indication of success [20]. This indication happens in two ways: (1) the reception of the confirmation POST message without EAP and with the AUTH option is an indication that the controller considers the EAP authentication finished. Second, the smart object knows that the EAP authentication went well if an MSK is available. Nevertheless, both entities still need to prove the possession of the MSK as mentioned in the EAP Key Management Framework (EAP KMF). It is worth noting that this last exchange (12–13) is integrity protected by an authentication tag embedded in an AUTH option. This provides a simple way of providing proof-of-possession of the MSK between the smart object and the controller.

To protect the communications between the smart object and the controller, after the LO-CoAP-EAP authentication service is completed, our solution is open to run the Security Association Protocol (SAP) for any particular LP-WAN technology, providing fresh, shared key material with the key derivation capabilities of the EAP KMF, as we elaborate in the next section.

The reader will note that there is a division of the exchanges in different phases in Figure 1. This division is there to compare easily the changes done over CoAP-EAP elaborated in Section 4.6, where we describe each phase and its function.

### 4.5. Bootstrapping Security Associations for LP-WAN

In this section, we specify how to derive key material from the MSK to secure the communications between the controller and the smart object. The derived keys are known as Transient Session Keys (TSKs) [21] in EAP lingo. Based on these keys, we can run virtually any security association that relies on pre-shared keys (in fact, existing wireless technologies such as WiFi or WiMAX already derive TSKs from the MSK). To illustrate this, we provide a simple example of this procedure based on LoRaWAN [6]. In particular, LoRaWAN requires the AppKey to run its security association protocol that involves two messages: join request/join response. Once the EAP authentication is successful, both the smart object and the controller share the MSK. From the MSK, we derive a TSK, which will be the AppKey in the LoRaWAN specification. For the derivation process, we use a similar Key Derivation Function (KDF) as the one specified in [46]. Specifically, we use AES Cipher-based Message Authentication Code (CMAC)-PRF-128 as the Pseudo Random Function (PRF), which uses AES-CMAC-128 as a primitive. Both primitives use AES-128 as the building block since it is widely used in constrained devices. Then, as the Key Derivation Function (KDF) we use the function PRF+ defined in [37], as recommended in [46]. The PRF+ is able to generate key material of different lengths. The example of the derivation of the AppKey can be seen in Equation (1). The AppKey is a 16-byte length key. The input for the KDF (PRF+) is the following: the MSK derived from the EAP method; an ASCII code representation of the non-NULL terminated string “IETF_LoRaWAN” (excluding the quotes), to which we concatenate the NULL value and the nonces exchanged. Sixty four is the length of the MSK; length is the length of the output (16 in the case of the AppKey).

(1) AppKey=KDF(MSK,″IETF_LoRaWAN″∣NULL∣nonce-c∣nonce-s,64,length)

The same KDF can be used to generate key material for any other technology that requires key material to protect data frames. The unique changes would be the replacement of the label “IETF_LoRaWAN” for another more appropriate one (e.g., IETF_SigFox) and the expected length of the key material.

**Figure 2:**
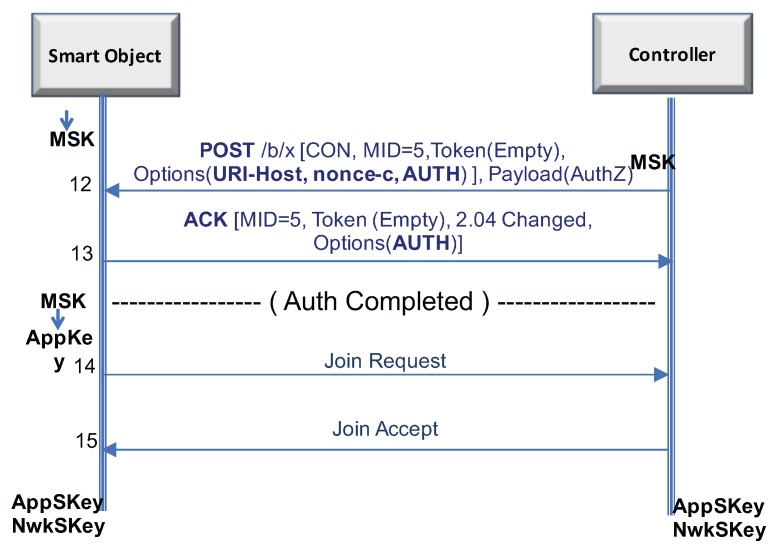
LoRaWAN security association establishment after LO-CoAP-EAP authentication.

As depicted in Figure 2, once both entities have derived the AppKey, they can simply run the LoRaWAN OTA security association protocol that allows one to derive two keys, Application Session key (AppSKey) and Network Session Key (NwkSKey), to protect communications (see more details in [6]).

### 4.6. Main Changes to CoAP-EAP

For the design of LO-CoAP-EAP, we perform a set of changes to CoAP-EAP to achieve the primary target of this paper: reduce the number of bytes dedicated to the network access authentication process traveling over the constrained link. The result is a reduction of the number of messages on flight and the size of the messages exchanged between the smart object and the controller.

To achieve this, we have analyzed each phase of CoAP-EAP in order to determine which parts can be simplified. In this sense, CoAP-EAP can be divided into five phases: (I) trigger; (II) exchange of nonces; (III) exchange of identity; (IV) exchange of EAP method; (V) sending the EAP success. Next, we see how LO-CoAP-EAP deals with each phase.

**Figure 3:**
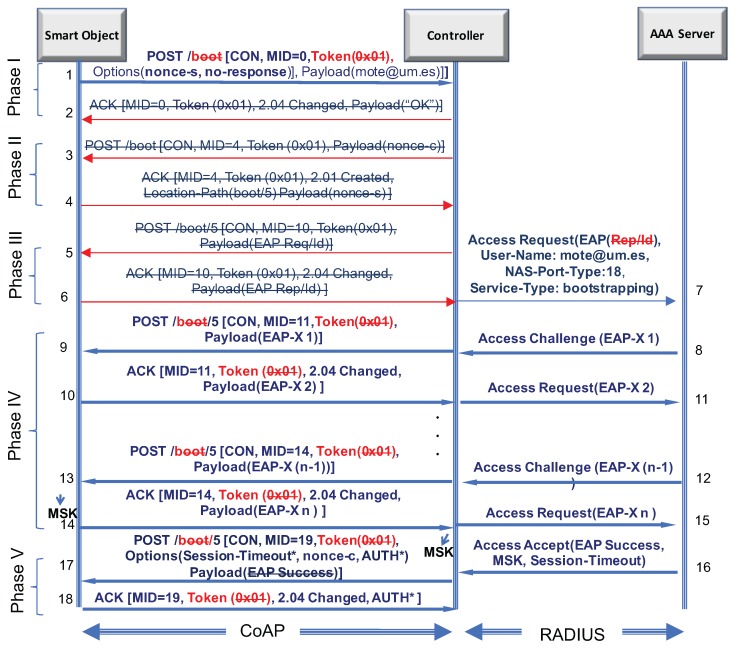
Changes to the original CoAP-EAP.

Figure 3 illustrates the changes in CoAP-EAP. The red (not highlighted) arrows signify that those messages are deleted in LO-CoAP-EAP, either because of being simplified or that information moved to other messages. The content in the messages is crossed out and colored in red, signifying that the content has been simplified. Common simplifications to all phases are: (1) the URI to identify the authentication service is reduced from /boot to /b to save three bytes in each request; (2) the token previously generated randomly, fixed for the duration of the exchange and used as session identifier is now set to empty. Discussion about the implications in LO-CoAP-EAP is shown in Section 4.7.1.

- Trigger (Phase I): To start the process in LO-CoAP-EAP, we send in the first POST used to trigger the authentication process the identity of the user in the payload of the message and the nonce-s in a new option (nonce option) for the controller. Additionally, this message carries a no-response option [42], which indicates to the controller that there is no need for a response of any kind to this request.
- Exchange of the nonces (Phase II): The nonce exchange that was previously done following the trigger message is avoided, and the nonces are embedded in other messages. Although this saves a complete exchange, its original purpose is still considered to alleviate Denial of Service (DoS) attacks, as is discussed further in Section 4.7.3.
- Exchange of the Identity (Phase III): After the nonce exchange, the EAP identity was requested. This exchange is now avoided, since the EAP standard specifies this exchange as optional. The identity of the user, as mentioned previously, is now sent in the trigger message. With this change, we save another exchange.
- EAP method exchange (Phase IV): The messages involved in the exchange of EAP method exchange are not further altered beyond the simplifications mentioned before and run as expected.
- Sending the EAP success (Phase V): Sending the EAP success message to the smart object is also optional. Avoiding sending the EAP success in the last exchange, we save a four bytes. We only send the nonce-c to the smart object for key derivation purposes in that last request.

It is worth noting that, as in any type of authentication process, we need radio communication between the smart object and the controller for the LO-CoAP-EAP service to work. Additionally, communication between the controller and the AAA infrastructure is also needed to complete the process. This communication is done through the WAN connection, not subject to the bandwidth limitations. Certainly, the EAP exchanges in the authentication involves an indirect communication between the smart object and the AAA server using the controller as the intermediary, but this process will only be done once; therefore, the smart object does not need constant connection with the AAA infrastructure after the authentication.

### 4.7. Additional Discussion

#### 4.7.1. Session Identifier and Empty Token

Changing the token value to empty saves some bytes sent over the link related to the authentication service. However, we argue that this change does not affect the operation of the protocol.

Indeed, the purpose of the CoAP token is to correlate a CoAP request and response [11]. More specifically, it is intended as a client-local identifier to differentiate between concurrent requests. Based on this, we state how the LO-CoAP-EAP protocol still maintains its functionality in spite of setting its value to empty. Firstly, since EAP is a lock-step protocol (see Section 2.2), the LO-CoAP-EAP protocol that transports EAP is also designed as lock-step. The reason is simple: to obtain network access through a specific controller, a single authentication process is enough. After all, the link is very constrained, and any traffic should be reduced. Therefore, the controller (CoAP client) will not send a new request until having received the corresponding response. Secondly, the original use of the token (as the session identifier) can be fulfilled by other means: we only need a known value by both parties that uniquely identifies the smart object. This can be done using the IPv6 address or the MAC address in case we consider a direct link between the smart object and the controller.

#### 4.7.2. Analyzing Retransmissions

Retransmissions are a tool to provide reliability for the communications, sending again a message if there is no indication that the message arrived successfully. The retransmission policy suggested in the CoAP standard has been adapted for LR-WPAN networks, though some improvements may be still performed in the default policy [11]. In particular, the default policy specifies to wait approximately 2 s between when a message is sent and the acknowledgment arrives and increasing exponentially the retransmission time to double the previous retransmission time, up to a maximum of four retransmissions.

Nonetheless, this retransmission policy needs further considerations in radio technologies with a very low bit rate, such as LP-WAN networks. For example, according to LoRa technology, assuming a Spreading Factor (SP) of 12, the bandwidth set to 125 kHz and the payload to the maximum allowed in LoRa (256 bytes) and setting the other variables as default in the Semtech LoRa Modem Calculator tool [47], a message will spend on the air approximately 7.5 s. As we can observe, before a CoAP message arrives at the other endpoint, a retransmission may be triggered (in some cases, more than one). Thus, it is necessary to adjust the retransmission policy in LPWAN networks.

We need to to assign the minimum elapsed time before a retransmission is sent. In CoAP, this is called ACK_TIMEOUT. Therefore, we need to consider the parameters used in LoRa when transmitting to get this number for the CoAP policy to establish its value. Basically, we need to know the Round Trip Time (RTT) of a message (the time it takes to arrive from a source to a destination and back) to establish ACK_TIMEOUT. Following the previous example and the implementation of the CoAP retransmission mechanism, we would set the ACK_TIMEOUT to 8 s (rounding up to cover processing time), which will give us a MAX_TRANSMIT_SPAN of 152 s, which corresponds with the time a client considers that a confirmable message was not received. Considering the results of the previous example, the controller will understand that a LO-CoAP-EAP authentication is discontinued after waiting 152 s for a piggybacked response.

LoRa technology has adaptive capabilities that need to be taken into account when establishing a retransmission policy for CoAP. As a side note, we comment that EAP has a retransmission mechanism that is disabled, since it is running over a our reliable lower layer LO-CoAP-EAP. Additionally, having a lower bit rate in the constrained link has an effect on the time each party involved in LO-CoAP-EAP needs to store any state related to it. The smart object, and its EAP state machine, needs to configure the amount of time to wait for a valid request before aborting (known as ClientTimeout in [48]). It is also the case for the AAA server that needs to keep the status related to the EAP authentication. If it is not stored for sufficient time, the AAA server might assume the authentication has failed, erasing the associated state, when it is simply just taking longer.

#### 4.7.3. DoS Attacks

The new design of LO-CoAP-EAP keeps the same security properties of CoAP-EAP discussed in Section 3.6 in [22], except for one particular aspect: LO-CoAP-EAP may save two messages after exchanging the smart object’s identity and use the smart object’s identity in the very first message (Step 1 in Figure 1), so that the controller can contact the AAA server immediately.

Although the modifications in the first message save bits in the link, this creates an additional state (authentication state) in the controller beyond just storing the smart object’s identity. In particular, this authentication state includes the EAP state machine, which must be initialized with different parameters [48], and the AAA client session required to communicate with the AAA server. This has an important implication: an attacker may blindly send the “trigger” message with different MAC or IPv6 addresses in a loop, therefore creating the authentication state for each trigger message sent to the controller.

When a link has unrestricted bandwidth, the number of messages starting authentications arriving at the controller may be very high. Each one will create some authentication state that the controller has to store for a determined time. This would provoke growth in the number of authentication states in the controller that surpass the capacity of the controller. However, if the capacity of the link is very reduced, then the maximum number of messages starting an authentication is dramatically reduced, as in the case of LP-WAN [49], limiting the authentication state generated in the controller. For example, if we use LoRaWAN, when the devices behave respecting the duty cycle, we can expect less than one authentication request per second. In the worst case, if an attacker were to use the channel at full capacity, we can expect around sixteen messages per second, in each channel. A controller that has to manage thousands of legitimate devices is assumed to be able to manage this amount of states created by attackers. In any case, if the controller evaluates that it is creating states at an abnormal rate, it can always perform the handshake to mitigate this effect. In summary, the link in LP-WAN is the main bottleneck and most restricted resource, which limits the number of authentications per second that can start in the controller. In fact, sending additional messages may create a problem in the link regardless of the use of LO-CoAP-EAP.

**Figure 4:**
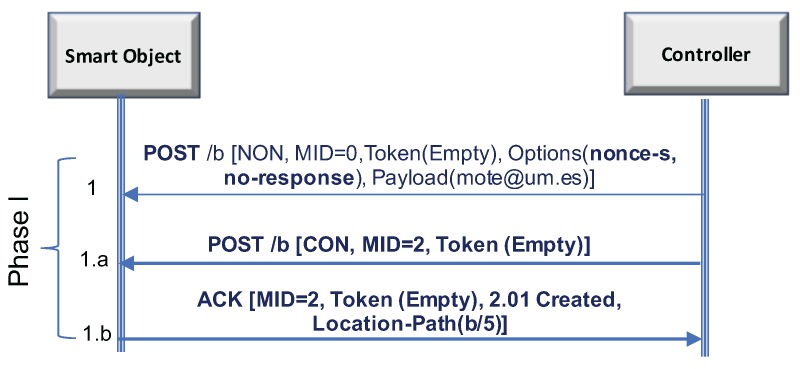
LO-CoAP-EAP handshake.

Either way, in order to alleviate potential DoS attacks, the controller can always engage in an optional (it was mandatory in CoAP-EAP) handshake (1a and 1b in Figure 4) with the smart object before creating the authentication state (EAP and AAA). In this manner, the attacker cannot provoke the creation of the authentication state using a loop since it must answer correctly the message sent by the controller. In any case, it is up to the controller’s policy to select when this handshake should be performed or not (which is out of scope of this work). For example, the controller may detect some irregular activity during the access (e.g., many triggers in a very short period of time) and, as a consequence, activate this handshake to avoid consuming resources for the authentication state.

## 5. Experimental Results

To test different technologies and conditions, we have performed evaluations in the Cooja simulator (Test-Bed 1) [50] and with real devices for LP-WAN (Test-Bed 2). The first testbed is used to achieve three goals: (1) to get an approximation of the performance of the protocol from one to several IP hops with different loss ratios, providing hard conditions in the link, since there is ongoing work in LP-WAN exploring the multi-hop case [51,52,53]; (2) to compare LO-CoAP-EAP with PANATIKI [38], which is a PANA implementation (a current standard for a link-layer independent network access authentication in IoT) adapted to the Contiki O.S.; and (3) to give proof that LO-CoAP-EAP also provides important improvement with respect to CoAP-EAP in LR-WPAN. Based on the results of this first approximation, we have prepared a real LP-WAN deployment using LoRa radio technology (a LoRaFabian [54] network), as a representative example of LP-WAN, with real devices for LP-WAN. Without loss of generality, we have obtained our results using a light and standard EAP method, EAP-PSK. This method consists only of four messages to complete the authentication. Since our solution is independent of the EAP method, any other method could have been used.

The parameters to be measured are: (1) message length, (2) network authentication time and success ratio (that is, the relation between finished and initiated authentications), (3) energy consumption and (4) memory footprint.

### 5.1. Message Length

In general, the message length is relevant in terms of the time the smart object takes to process it (including the time to send and receive messages over the network). In LP-WAN, this is especially relevant, taking into account the restrictions of LP-WAN in the link.

Table 2 shows the comparison in terms of message length (in bytes) for each alternative: (1) PANATIKI, (2) CoAP-EAP, (3) LO-CoAP-EAP with handshake and (4) LO-CoAP-EAP without handshake. We detail the length of the EAP lower layer without the EAP message (LL) and including it (LL + EAP). PANATIKI is shown for reference since a detailed description of the message length comparison with CoAP-EAP is done in [22]. This will give a better understanding of the impact the redesign has on the reduction of the size of the protocol and, overall, the percentage of bytes saved in each case.

Overall, an important reduction in size of the lower layer can be appreciated. With CoAP-EAP over PANATIKI, we reduce up to ≈50% comparing the lower layer and up to ≈32% the lower layer + EAP messages. With LO-CoAP-EAP over PANATIKI, we reduce up to ≈70% compared with the lower layer, and up to ≈50% the lower layer + EAP messages. Comparing LO-CoAP-EAP with CoAP-EAP and with LO-CoAP-EAP with handshake, we have a reduction of ≈30% for the lower layer and a reduction of ≈22% over the whole protocol exchange (lower layer + EAP messages). For LO-CoAP-EAP with handshake, this reduction is ≈39% for the lower layer and ≈25% for the whole protocol exchange compared with CoAP-EAP. This reduction is important to consider in very constrained links such as LP-WAN networks, as we show in Section 5.3.

### 5.2. LO-CoAP-EAP Performance in the Cooja Simulator

**Figure 5:**
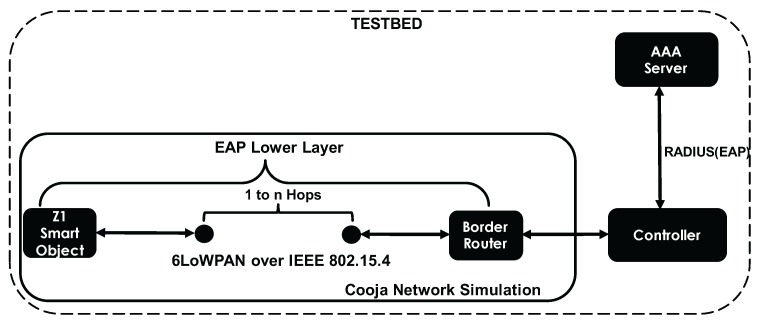
Cooja test-bed.

Figure 5 shows the test-bed we use in the Cooja Network Simulator with Contiki OS Version 2.7 [50]. The smart objects used are the Zolertia Z1 with 92 kB of nominal ROM when compiled with 20-bit architecture support and 8 kB of RAM. The compiler is msp430-gcc Version 4.7.2. The specifications of the computer used for running the test-bed are shown in Table 3. In terms of the software packages, we have used cantcoap [55] ported to C language since it gives us the flexibility needed to only create CoAP messages without including the REST engine integration, which is sufficient for our proof-of-concept implementation. In this test-bed, we use the RPL border router, which enables communication between the simulated Cooja Network and the outside physical network where the controller is located. Between the border router and the smart object, there can be zero or more smart objects. Having several hops between the smart object and the controller allow us to observe the behavior of the network access authentication process in multi-hop networks and how each parameter is affected (network authentication time, success ratio and energy consumption), when intermediate smart objects act as IP-forwarders. Following the recommendations in [56], we have performed the simulations in Cooja with a randomly generated seed to automate running the simulations and have used the default values for Radio Duty Cycling (RDC) in Cooja: the contikimac_driver RDC driver with a channel check value of 8 Hz.

We show the different performance measurements gathered with the Cooja simulator to compare LO-CoAP-EAP performance with CoAP-EAP and PANATIKI. The evaluation is done in different scenarios: (1) with different numbers of hops ranging from 1–4 and (2) with different lossy environments with loss ratios of 0.0 0.1 and 0.2. These data have been gathered after performing around 100 authentications per scenario.

#### 5.2.1. Network Authentication Time in Cooja

**Figure 6:**
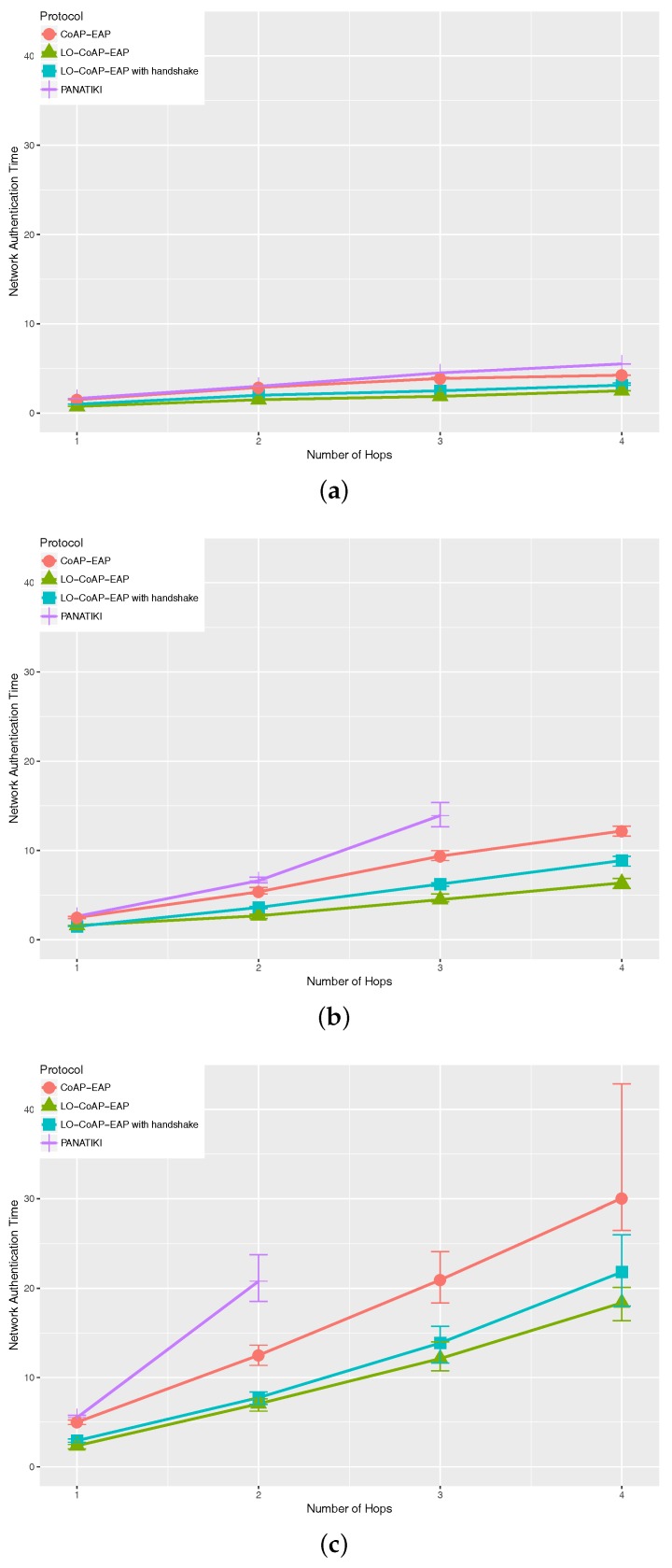
Median network authentication time in Cooja for LO-CoAP-EAP, CoAP-EAP and PANATIKI. (**a**) Median network authentication time with 0.0 loss ratio; (**b**) median network authentication time with 0.1 loss ratio; (**c**) median network authentication time with 0.2 loss ratio.

Figure 6 shows the network access authentication time. Generally, we can see that PANATIKI sets an upper bound to the authentication time, while LO-CoAP-EAP with handshake sets the lower bound. All CoAP-based solutions show a statistically significant different with respect to PANATIKI.

As we can see in Table 4, the improvement is up to ≈68% comparing any CoAP-based solution with PANATIKI. As expected, this difference is partially due to the reduction in message length of the CoAP-based solutions and the reduction of the number of messages in LO-CoAP-EAP. Since PANA has longer messages, PANATIKI takes longer to complete an authentication than any CoAP-based solutions. Among the CoAP based solutions, LO-CoAP-EAP improvement ranges from ≈26–≈53% with respect to CoAP-EAP, also due to the reduction in size and number of exchanges in LO-CoAP-EAP.

With CoAP-EAP, there was little difference with PANATIKI in the most favorable conditions (fewer hops and loss ratio), whereas with LO-CoAP-EAP with or without handshake, the improvement is noticeable, even with more favorable conditions.

**Figure 7:**
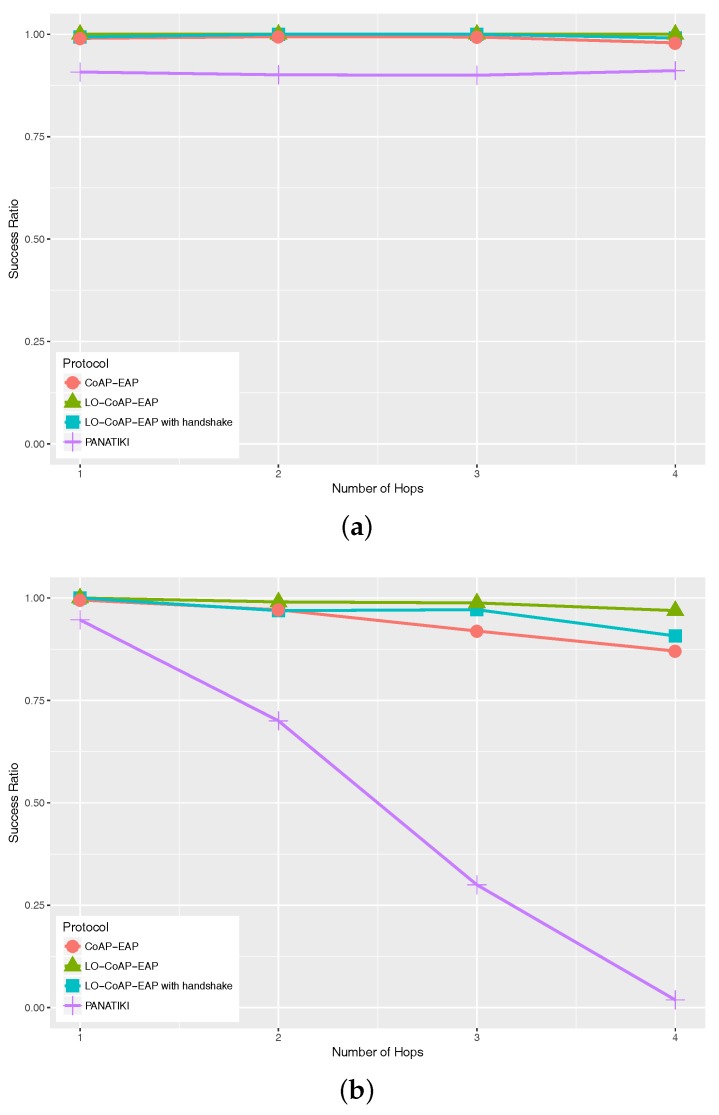

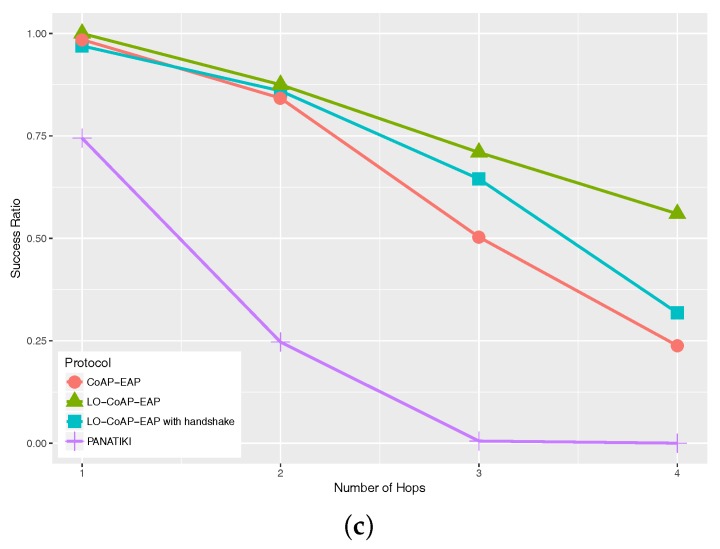
Success ratio in Cooja for LO-CoAP-EAP, CoAP-EAP and PANATIKI. (**a**) Success ratio with 0.0 loss ratio; (**b**) success ratio with 0.1 loss ratio; (**c**) success ratio with 0.2 loss ratio.

Figure 7 shows the success ratio with different loss ratios: 0.0 Figure 7a, 0.1 Figure 7b and 0.2 Figure 7c. When the packet loss ratio increases, the possibility of completing a bootstrapping procedure decreases. In this sense, we can see that PANATIKI sets a lower bound to the success ratio, while LO-CoAP-EAP sets an upper bound. CoAP-based solutions demonstrate a better performance in every packet loss ratio in comparison with PANATIKI. As mentioned before, the length and quantity of messages to exchange play an important role, not only in the time it takes to complete an authentication, but also to complete the authentication successfully.

As can be seen, PANATIKI is not able to finish authentications with three hops. Table 5 shows that the improvement ranges from ≈5–100% comparing any CoAP-based solution with PANTIKI. CoAP-based solutions are able to complete the authentication generally with a greater success ratio, since the reduction in the number of messages and their shorter message length has an impact on the overall number of exchanges and a reduction in fragmentation. Among the CoAP-based solutions, in the more favorable cases, the improvement is negligible and increases as the conditions are more severe. LO-CoAP-EAP improvement with respect to CoAP-EAP goes up to 43%. This difference can also be attributed to the reduction in message length and the number of messages.

When evaluating the time to complete the network access authentication, it is worth noting that this process is done prior to the smart object being able to send or receive data traffic. This means that how much time it takes to finish the authentication is not so important. However, it is important to finish it even in harsh conditions. This is even more relevant in LP-WAN where the time to transmit and receive messages is considerable. For example, with a 0.2 loss ratio and four hops, LO-CoAP-EAP takes ≈18 s to complete the network access authentication. This time is reasonable because: (1) we are assuming a very constrained link; (2) this process is only done once, before the smart object can do anything else; (3) it will only be done again in case the device looses its state or it resets.

#### 5.2.2. Energy Consumption in Cooja

To perform the evaluation of the energy consumption, we used the Powertrace [57] tool that comes with the Cooja simulator. We use it to estimate the median energy consumed by each network authentication (mJ/network authentication) in LO-CoAP-EAP and CoAP-EAP (PANATIKI is also set as the reference). There are different measurements: CPU consumption when the smart object is fully operative (not in low power or sleeping mode); the consumption when transmitting (TX), receiving (RX) and the total energy consumption. For the sake of simplicity, we show the total energy consumption per authentication as a representative measurement, since it gives a general view of the requirements of each solution in terms of energy consumption. The total energy consumption is measured for three different loss ratio scenarios; 0.0, 0.1 and 0.2, respectively; and for different hops ranging from 1–4 hops between the smart object and border router.

**Figure 8:**
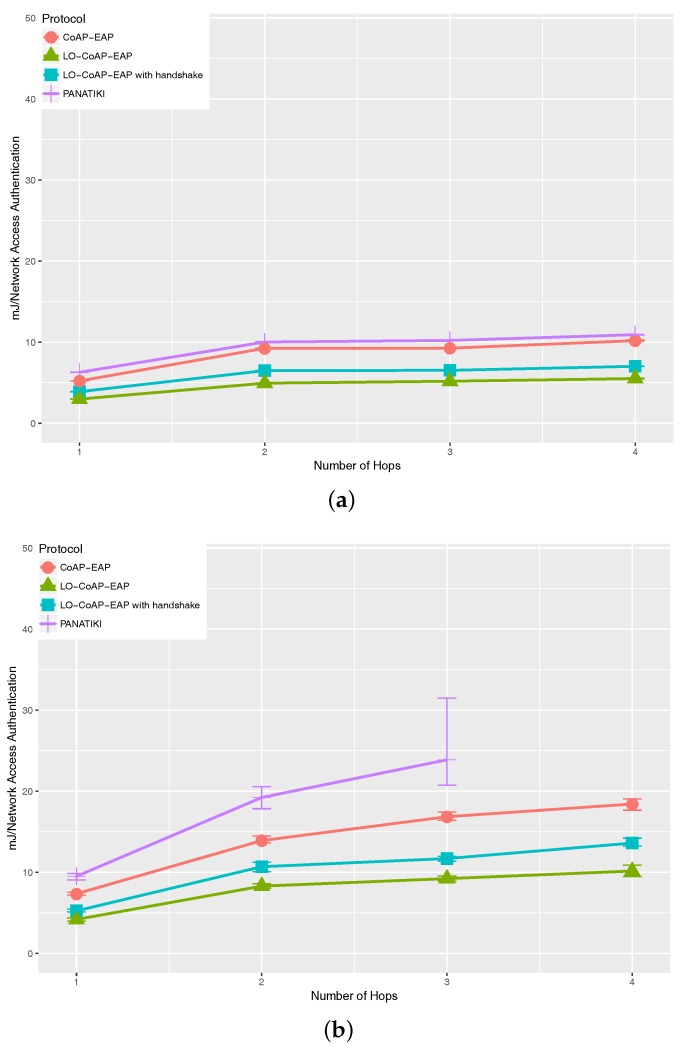

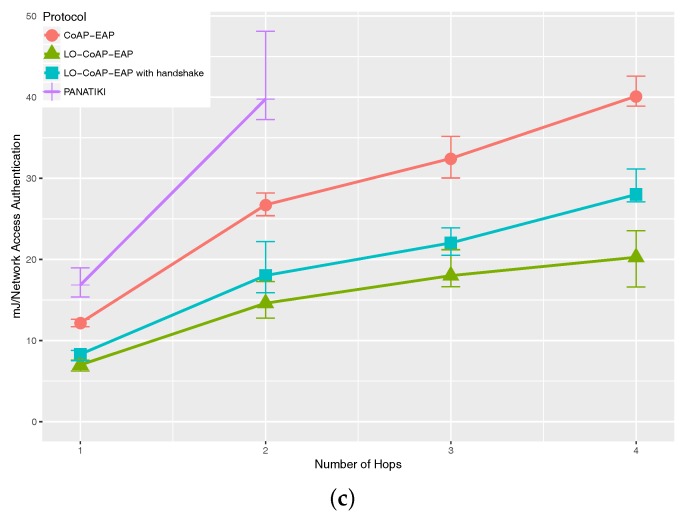
Total energy consumption in Cooja for LO-CoAP-EAP, CoAP-EAP and PANATIKI. (**a**) Total energy consumption with a 0.0 loss ratio; (**b**) total energy consumption with a 0.1 loss ratio; (**c**) total energy consumption with a 0.2 loss ratio.

Figure 8 shows the energy consumption with different loss ratios: 0 Figure 8a, 0.1 Figure 8b and 0.2 Figure 8b. The energy consumption, is greatly affected by the energy dedicated to sending and receiving messages. As the number and length of the messages increase, the energy consumption increases, as well. This is aggravated by the fragmentation of the messages, the retransmissions, etc.

The improvement, as shown in Table 6, ranges from 7–63% comparing any CoAP-based solution with PANTIKI. The greater the length of the messages, more bytes are sent over the network. This is worsened when fragmentation occurs in a message, hindering the completion of an authentication. In the particular case of PANATIKI, it also has a more aggressive retransmission policy than CoAP, which results in sending more traffic over a constrained network. Among the CoAP-based solutions, LO-CoAP-EAP improvement ranges from 23–50% with respect to CoAP-EAP. The reduction in size and number of messages is also a factor in the reduction of the energy consumption of LO-CoAP-EAP over COAP-EAP. This is also noticeable comparing LO-CoAP-EAP with and without handshake. The added exchange of the handshake also influences significantly the energy consumption, as can be appreciated in Figure 8.

#### 5.2.3. Memory Footprint in Cooja

Table 7 shows the memory footprint of each implementation. First, we show for reference the memory footprint of an empty program, representing the memory use of the O.S. After that, we show the memory footprint of each solution that includes the empty program measurements. The two columns represent how much memory is used in both ROM and RAM. The empty program uses ≈20 kB of ROM and ≈3.4 kB of RAM; PANATIKI uses ≈47 kB of ROM and ≈6 kB of RAM; CoAP-EAP ≈47.5 kB of ROM and ≈5.5 kB of RAM; and LO-CoAP-EAP ≈48 kB of ROM and ≈5.5 kB of RAM. These values include the O.S., the necessary network modules, the EAP state machine, the EAP method and the EAP lower layer. For a fair comparison, we use the same EAP state machine and EAP method.

Comparing the results, we can say that all EAP lower layers have a similar memory footprint. In PANATIKI, the O.S. employs ≈43.5% of ROM and 57% of RAM. For both CoAP-EAP and LO-CoAP-EAP, the S.O represents ≈43% ROM and ≈62% of RAM. The differences between CoAP-EAP and CoAP-EAP are in terms of code, to handle the case where it is handshake or not. Even though the CoAP-based solutions use more memory dedicated to the code, this is not an issue, since by using CoAP, we are sharing a common library present in most IoT devices (a CoAP implementation). PANATIKI would have to add the CoAP library to the existing code to support CoAP services, and this is one of the advantages of using a CoAP-based EAP lower layer for IoT (see more details in [22]).

### 5.3. LoRaFabian Network Test-Bed

**Figure 9:**
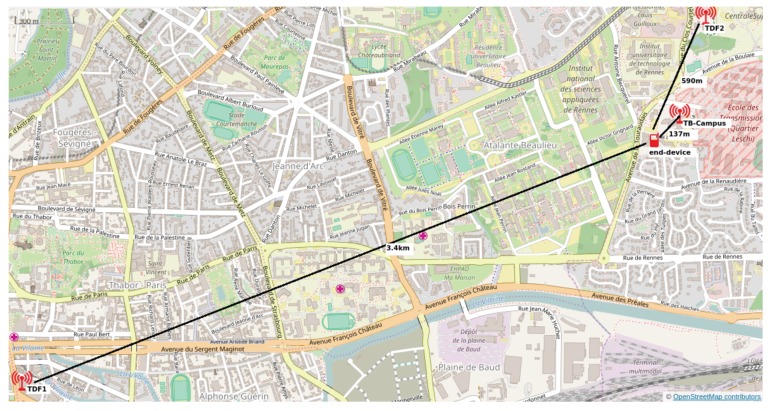
LoRaFabian network in Rennes, France.

For the test with LP-WAN, we use a real LP-WAN deployment: the LoRa network in Rennes, France. A snapshot of part of the city of Rennes where the deployment is located is shown in Figure 9. The deployment consists of three antennas (LoRa base stations) from Kerlink covering a fair portion of the city. Two of the antennas are installed on the structures maintained by TDF (Telediffusion de France), the company that provides telecommunication services in France, and the closest antenna is the one that is installed in the IMTatlantique’s campus itself (previously known as Telecom Bretagne). The location of the measurement where the end device was used is more than 100 m away from the antenna in IMT atlantique.

**Figure 10:**
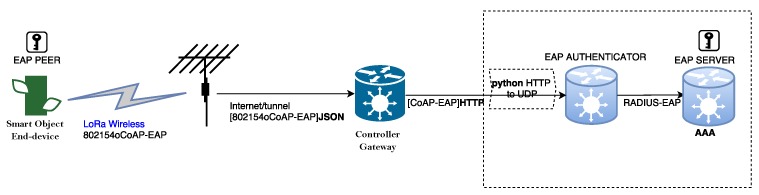
Architecture of the LoRaFabian network.

The setup is shown in Figure 10. It has a star topology, which means the end nodes can reach the gateway in a single hop. The smart object instantiated in the end-device (from froggy factory (www.froggyfactory.com)) runs on Contiki and has an embedded LoRa radio coupled with an Arduino board to derive its power.

According to the architecture presented in Section 4.3, the controller will be in the gateway. Because it was not possible to avoid disrupting the production deployment, the controller was located in an external entity. For the gateway to communicate with the external authenticator, the CoAP messages were sent over HTTP to the controller that has a Python hook enabled that serves as a proxy to transfer the CoAP packets from HTTP to UDP. Although separated, in this test-bed, the controller and gateway would be co-located in the same entity in a production deployment. Finally, the AAA server runs FreeRADIUS Version 2.0.2 (freeradius.org).

In this test-bed, the nearest antenna is located <100 m from the end-device. These data have been gathered after performing 15 authentications per parameter to obtain network access authentication time and energy consumption.

The LoraServer sends a beacon every 30 s, which is broadcast to the network using the antenna. The end device, upon receiving the beacon from the antenna, shall register itself to the network by sending a response to the beacon with its hardware address after a random delay in order to avoid collision with other devices. The network shall send a message to the devices by specifying the device’s hardware address as the destination address in the 802.15.4 frame. The end device is continuously listening on the channel frequency except for the period of transmission.

#### 5.3.1. Network Authentication Time in LoRa

**Figure 11:**
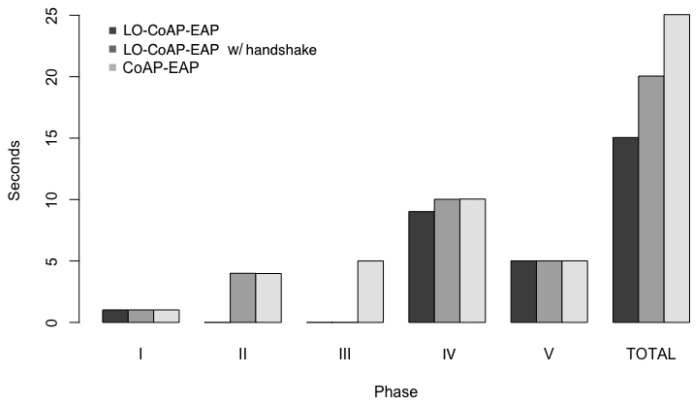
Network authentication time.

A measurement that characterizes each solution of network authentication is the time it takes to complete. The graphs showing the median network authentication times can be seen in Figure 11. As may be appreciated in those figures, we can say that there is a statistically significant improvement in the network authentication time in LO-CoAP-EAP over CoAP-EAP.

To measure the network authentication, we have to consider that the Round Trip Time (RTT) constitutes the major portion of the network access authentication time in this test-bed. It is the time that is measured between two successive message received by the end device from the LoRa antenna, and it includes the travel time over the air, the message processing time in the Python hook, the authenticator and the RADIUS server. The total network authentication time is the interval that is measured between the time when the end device sends the first message to trigger the authentication mechanism (Phase I) and the time when the ACK for the last request containing the AUTH option is sent for the key confirmation (Phase V). Figure 11 shows the time taken by the end devices at each phase and the total time of the authentication process for one instance of each of the solutions (CoAP-EAP, LO-CoAP-EAP and LO-CoAP-EAP with handshake).

We can see that at Phase I, all solutions spend a similar time to complete this task. After that, CoAP-EAP and LO-CoAP-EAP with handshake show a similar time as a consequence of the handshake exchange. On the contrary, LO-CoAP-EAP saves this time. Regarding Phase III where the EAP identity is exchanged, only CoAP-EAP engages in that exchange. Phase IV, common to all solutions, where the EAP method is exchanged, presents similar values in the time spent during the exchange. Finally, for the final exchange, where the AUTH option is present, we can also see similar values for all solutions.

As we can see, LO-CoAP-EAP has fewer exchanges and less bytes traveling in the network, and for these reasons, it takes lesser time (15 s) to complete the network access authentication as compared to the CoAP-EAP (25 s). LO-CoAP-EAP with handshake takes 20 s, an improvement of 5 s over CoAP-EAP, which is still a considerable improvement. Thus, we have achieved a reduction of ≈20% in the authentication time for LO-CoAP-EAP performing the handshake and a reduction of ≈40% in time for LO-CoAP-EAP. The question if this is a reasonable time has to take into account that this process is done one time, before the device can access the network. This is a necessary step to secure the communications and manage the network. Furthermore, the limited bandwidth in LP-WAN (LoRa in this case) bound longer transmission times, so we think that these times are not unusual.

As a side note and apart from the differences with the Cooja simulations, we can see that we have similar values in LP-WAN to the worst cases in Cooja (with a 0.2 loss ratio and 3–4 hops). This gives us an approximation of the harsh conditions LP-WAN networks are dealing with to transmit data over the network.

#### 5.3.2. Energy Consumption in LoRaFabian

**Figure 12:**
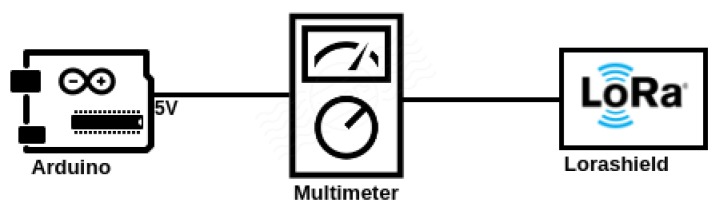
Energy measurement setup.

As there is no Powertrace application support for the LoRa platform, we have used a digital multimeter to measure the current drawn by the shield containing the smart object, as in Figure 12 during the network authentication process.

The smart object is in idle mode until it receives the first radio packet and continues to work in radio mode throughout the network authentication process.

The parameters used to measure the energy consumption are the following: the nominal power for operation is 5 V; the current consumption in each mode is: 15 mA when idle and 19.6 mA in transmission (TX) and reception (RX). With these values, we measure the energy consumption of each solution.

Table 8 shows the energy consumption of the test done in LoRa. Comparing the energy consumption of the solutions and taking as reference CoAP-EAP, we appreciate a reduction of ≈20% in energy consumption for LO-CoAP-EAP performing the handshake and a reduction up to ≈40% in energy consumption for LO-CoAP-EAP. This clearly shows a considerable improvement over the previous solution.

#### 5.3.3. Memory Footprint in LoRaFabian

Table 9 shows the memory use for the implementations. For these experiments, we use the STM32F103RB mote, which as 128 kBytes of ROM and 20 kBytes of SRAM. For reference, we show the memory footprint of an empty program and after that the values of CoAP-EAP and LP-CoAP-EAP. The empty program uses ≈8 kB of ROM and ≈2.6 kB of RAM. Both CoAP-based solutions have a similar memory footprint; CoAP-EAP ≈40.7 kB of ROM and ≈8.7 kB of RAM; LO-CoAP-EAP ≈41.1 kB of ROM and ≈8.7 kB of RAM.

The empty program is an estimate of the memory footprint of the O.S. CoAP-EAP and LO-CoAP-EAP include, additionally, the necessary network modules, the EAP state machine, the EAP method and the EAP lower layer. The EAP state machine and EAP method are the same in all instances. Comparing the results, we can say that for both CoAP-EAP and LO-CoAP-EAP, the O.S. represents ≈20% ROM and ≈33% of RAM. The differences between CoAP-EAP and LO-CoAP-EAP are in terms of code, due to the changes to manage the situation with handshake.

## 6. Conclusions and Future Work

In this work, we have highlighted that the inclusion of new radio technologies into the IoT landscape, known as LP-WAN, has posed an interesting challenge, when network access authentication is taken into account. LP-WANs pose even more restricted links than those known in LR-WPAN. In particular, besides the existing constraints in LR-WPAN, in terms of resources such as memory, CPU and energy consumption, the bandwidth is severely restricted, as well.

We have presented LO-CoAP-EAP, a complete redesign of a previous solution for network access authentication (CoAP-EAP), to cope with the restrictions imposed by LP-WAN. LO-CoAP-EAP has been designed to further reduce the number and the length of messages to deal with the very limited LP-WAN bandwidth. LO-CoAP-EAP still uses CoAP, EAP and AAA to provide scalability, flexibility and wireless independence, however reducing the number and length of the messages. Moreover, once the network access authentication is finished successfully, it is possible to provide key material to different types of LP-WAN security associations to protect the access to the network.

To assess the performance of LO-CoAP-EAP and to show how it overcomes previous work, we have performed simulations with the Cooja network simulator to confirm that LO-CoAP-EAP improves CoAP-EAP and PANA in the context of LR-WPAN. Not only that, we have run LO-CoAP-EAP and CoAP-EAP over a real LoRa network, the LoRaFabian network, as a representative example of LP-WAN, to experimentally prove that the LO-CoAP-EAP provides an improvement in both LP-WAN and LR-WPAN networks. It is worth noting that further improvements are expected in the performance once CoAP is adapted for LP-WAN networks, as is currently happening in the context of the IETF [10]. Our design will not change, but CoAP will provide an even more reduced EAP lower layer, which will further improve the results obtained.

Future work has been planned to cover the case where the smart object has no direct communication with the controller before the authentication. For example, the smart object may not have any routable IP address to reach the controller until it is authenticated and authorized to join the network, for instance as happens in ZigBee networks. In this case, the assistance of a node belonging to the network that intermediates between the smart object and the controller is required to perform the network access authentication. Thus, a CoAP-based intermediary may need to be designed and implemented to perform an authentication in these cases.

## Figures and Tables

**Table 1:** Crypto suites used by LP-WAN technologies to authenticate and encrypt the frames.

Technology	Authentication/Integrity Algorithm	Payload Encryption Algorithm	Keyl Management Protocol
LoRaWAN	AES-128 Cipher-based Message Authentication Code (CMAC)	AES-128	YES
Sigfox	N/A	N/A	NO
IEEE802.15.4k	N/A	N/A	-
IEEE802.15.4g	AES­-CCM	AES­-CCM	-
Random Phase Multiple Access (RPMA)	Two­way16­byte-hash	256-bit	-
DASH-7	AES Cipher Block Chaining(CBC)-MAC-128/64/32 AES-CCM-128/64/32	AES Counter Mode (CTR)	-
Weightless	AES-128/256	AES-128/256	YES
NB-LTE	Authentication and Key Agreement (AKA)(128 bit keys)	AKA (128 bit keys)	YES

**Table 2:** Comparison of the message lengths. PANATIKI, PANA implementation of Contiki.

		PANATIKI		CoAP-EAP		LO-CoAP-EAP with Handshake		LO-CoAP-EAP without Handshake	
**Phase**	**Message (CoAP-EAP)**	**LL**	**LL + EAP**	**LL**	**LL + EAP**	**LL**	**LL + EAP**	**LL**	**LL + EAP**
I	1) POST	16	16	13	13	29	29	29	29
II	2) POST(nonce-c)	40	40	18	18	6	6	-	-
	3) ACK(nonce-s)	40	40	20	20	8	8	-	-
III	4) POST(Request/Id)	43	48	16	21	-	-	-	-
	5) ACK(Reponse/Id)	41	60	9	28	-	-	-	-
IV	6) POST(EAP-PSK 1)	27	56	16	45	9	38	7	36
	7) ACK(EAP-PSK 2)	24	84	9	69	5	65	9	69
	8) POST(EAP-PSK 3)	25	84	16	75	9	68	9	68
	9) ACK(EAP-PSK 4)	25	68	9	52	5	48	5	48
V	10) POST(EAP success)	84	88	35	39	34	38	34	38
	11) ACK	52	52	27	27	23	23	23	23
**% Reduction over PANATIKI**		-	-	≈55%	≈36%	≈69%	≈49%	≈72%	≈51%
**% Reduction over CoAP-EAP**		-	-	-	-	≈32%	≈20%	≈38%	≈23%
**Total**		417	636	188	407	128	323	116	311

LL: Lower Layer message length; LL + EAP: Lower Layer message length including EAP message length.

**Table 3:** Cooja test-bed specifications.

CPU	Intel(R) Core(TM) i5-2400 CPU @ 3.10 GHz
RAM	4 GiB DIMM DDR3 Synchronous 1333 MHz
O.S.	Ubuntu Server 12.04.5 LTS-32 bits
Kernel	3.13.0-32-generic

**Table 4:** Comparing the improvement of network authentication Time among PANATIKI, CoAP-EAP, LO-CoAP-EAP without handshake and LO-CoAP-EAP with handshake.

Protocol	CoAP-EAP	LO-CoAP-EAP with Handshake	LO-CoAP-EAP without Handshake
**PANATIKI**	**4.5–39.9%**	**33.6–62.8%**	**38.8–67.7%**
**CoAP-EAP**	**-**	**26.5–41.3%**	**35.5–52.8%**
**LO-CoAP-EAP with handshake**	**-**	**-**	**0–28.2%**

**Table 5:** Success ratio comparison.

Protocol	CoAP-EAP	LO-CoAP-EAP with Handshake	LO-CoAP-EAP without Handshake
**PANATIKI**	**4.9–100%**	**5.4–100%**	**5.4–100%**
**CoAP-EAP**	**-**	**0–25.2%**	**0.5–57.5%**
**LO-CoAP-EAP with handshake**	**-**	**-**	**0–43.2%**

**Table 6:** Energy consumption comparison.

Protocol	CoAP-EAP	LO-CoAP-EAP with Handshake	LO-CoAP-EAP without Handshake
**PANATIKI**	**6.7–32.8%**	**35.3–54.7%**	**49.3–63.3%**
**CoAP-EAP**	**-**	**23.2–32.6%**	**40.4–49.5%**
**LO-CoAP-EAP with handshake**	**-**	**-**	**15.8–27.7%**

**Table 7:** Memory footprint of the implementations in Cooja.

Implementation	ROM (bytes)	RAM (bytes)
**Empty Program**	20,505	3410
**PANATIKI**	47,151	6006
**CoAP-EAP**	47,601	5484
**LO-CoAP-EAP**	48,019	5484

**Table 8:** Energy measurement in LoRaFabian.

**CoAP-EAP network authentication**	2453.92 mJ/authentication
**LO-CoAP-EAP (with handshake) network authentication**	1964.9 mJ/authentication
**LO-CoAP-EAP (without handshake) network authentication**	1473.92 mJ/authentication

**Table 9:** Memory footprint of the implementations in LoRa.

Implementation	ROM (bytes)	RAM (bytes)
**Empty Program**	7908	2596
**CoAP-EAP**	40,744	8668
**LO-CoAP-EAP**	41,144	8668

## Abbreviations

The following abbreviations are used in this manuscript:

6LoWPAN	IPv6 over Low power Wireless Personal Area Networks
AAA	Authentication, Authorization and Accounting
AES	Advanced Encryption Standard
CoAP	Constrained Application Protocol
DTLS	Datagram Transport Layer Security Version
EAP	Extensible Authentication Protocol
EAP KMF	EAP Key Management Framework
IEEE	Institute of Electrical and Electronics Engineers
LP-WAN	Low-Power Wide-Area Network
LR-WPAN	Low-Rate Wireless Personal Area Network
LoRa	Long Range
MSK	Master Session Key
NAS	Network Access Server
OTA	Over The Air
PANA	Protocol for Carrying Authentication for Network Access
PSK	Pre-Shared Key
RADIUS	Remote Authentication Dial In User Service
RPL	Routing Protocol for Low-Power and Lossy Networks
SAP	Security Association Protocol
TSK	Transient Session Key
URI	Uniform Resource Identifier
